# Vitamin D supplementation and risk of stroke: A meta-analysis of randomized controlled trials

**DOI:** 10.3389/fneur.2022.970111

**Published:** 2022-08-18

**Authors:** Jia Fu, Junfang Sun, Chao Zhang

**Affiliations:** ^1^Department of Neurology, Chifeng Municipal Hospital, Chifeng, China; ^2^The Second Affiliated Hospital of Baotou Medical College, Inner Mongolia University of Science and Technology, Baotou, China; ^3^Department of Geriatrics, Chifeng Municipal Hospital, Chifeng, China

**Keywords:** stroke, vitamin D, meta-analysis, prospective randomized controlled trials, 25-hydroxy vitamin D

## Abstract

**Background:**

Previous observational studies have supported the hypothesis that vitamin D supplementation protects against stroke. However, several current intervention studies contradict this observation. Therefore, we conducted a meta-analysis to investigate further the association between vitamin D supplementation and the risk of stroke.

**Methods:**

This meta-analysis was conducted in accordance with the PRISMA statement and included all the randomized controlled trials (RCTs) that analyzed the relationship between vitamin D supplementation and the risk of stroke. A literature search strategy was established, and the following Medical Search Terms (MeSH) were used: “vitamin D,” “Calcitriol,” “Calcifediol,” “Cholecalciferol,” “25-Hydroxyvitamin D 2,” “ergocalciferols,” “stroke,” and stroke-derived terms. We searched for articles published before January 2022 in several databases, namely, PubMed, Web of Science, EMBASE, and The Cochrane Library. We also reviewed references included in relevant published meta-analyses and searched the http://www.ClinicalTrials.gov website for additional RCTs. The *Q* test and *I*^2^ were utilized to assess the degree of heterogeneity among the studies. Review Manager 5.3 and STATA16.0 software programs were used to assess the literature quality and perform statistical analyses.

**Results:**

In total, twenty-four RCTs (86,202 participants) were included. There was no statistical heterogeneity among the RCTs (*I*^2^ = 0.0%, *P* = 0.94) included in this meta-analysis. We determined that vitamin D supplementation was not associated with a reduced risk of stroke compared with the placebo (RR = 1.02, 95% CI: 0.93–1.13, *P* = 0.65). In total, 10 studies only included women, and 14 studies included women and men among the 24 RCTs. Therefore, we performed a subgroup analysis based on sex. After the subgroup analysis, the effect remained statistically insignificant (mixed-sex group: RR = 1.06, 95% CI: 0.93–1.22, *P* = 0.37, women group: RR = 0.98, 95% CI: 0.86–1.13, *P* = 0.80). The results were generally comparable, based on age, body mass index (BMI), follow-up period, baseline 25-hydroxyvitamin D (25(OH)D) levels, the designated endpoint, latitude, vitamin D dosage, type of vitamin D administered, and an absence or presence of concurrent calcium supplementation (*P* > 0.05).

**Conclusion:**

Our study revealed that additional vitamin D supplementation did not reduce the risk of stroke. Therefore, additional RCTs of similar design should not be encouraged to assess any association between vitamin D supplementation and reduced stroke risk.

## Introduction

Stroke is the second leading cause of death worldwide, resulting in more than 5.7 million deaths annually (1). In addition, stroke is the primary cause of disability in adults, generating substantial economic costs for treatment and long-term care of stroke victims (1, 2). The burden of stroke has increased annually because of the increasing aging of the population and the increase in corresponding risk factors of poor diet, obesity, physical inactivity, and tobacco use. Low- and middle-income countries (LMICs) are experiencing a growing burden of these major behavioral risk factors compared with the high-income countries (HICs). Residents in LMICs have a higher risk of a severe adverse health event such as myocardial infarction or stroke, due to the limited capacity to detect cardiovascular, respiratory, or related disorders (CVRDs) and provide early treatment (3). Therefore, effective stroke prevention strategies are urgently needed.

Vitamin D has attracted considerable attention as a possible treatment for stroke prevention. It is an essential substance that includes both cholecalciferols and ergocalciferols in the human body and plays a vital role in regulating the homeostasis of calcium and phosphates (4). Vitamin D can be formed in the skin by the action of ultraviolet rays upon its precursors, 7-dehydrocholesterol, and ergosterol (4). It is known that Vitamin D produces various effects on obesity, energy expenditure, and pancreatic cell activity by activating its nuclear receptor in vascular endothelial and myocardial cells and regulating the renin–angiotensin–aldosterone system (5). Vitamin D also exhibits several neuroprotective effects (6–8), namely, enhancing synaptic plasticity (9), reducing oxidative stress (10), and reducing brain damage and inflammatory responses in ischemic and neurodegenerative diseases (11, 12). Several studies have demonstrated that vitamin D deficiency was an independent risk factor for ischemic stroke (13–15). Vitamin D deficiency has been associated with increased severity and poor prognosis following stroke (7, 8, 16). Vitamin D deficiency is also thought to be associated with arteriosclerosis; vascular dysfunction, left ventricular hypertrophy; and reduced metrics for diabetes, hypertension, and hyperlipemia, all of which are associated with the incidence of stroke (17).

It is known that vitamin D deficiency is widespread in different latitudes worldwide, especially in China, the Middle East, Mongolia, and India. More than 50% of the world population has serum 25(OH)D levels below 50 nmol/L, at least during the winter (18). Thus, the possibility of reducing the risk of stroke with vitamin D supplementation has been a research focus for some time. If this hypothesis could be confirmed, vitamin D supplementation could be an economical, safe, and widely available approach to reducing the risk of stroke (17, 19). Several previous prospective population health studies have shown that dietary intake of vitamin D has reduced the incidence of and mortality from stroke in the middle-aged and older adults (20, 21). However, the results of RCTs on the association between vitamin D supplements and the prevention of cardiovascular disease, including stroke showed no protective effect of vitamin D supplementation against stroke (22–25). Furthermore, meta-analyses on vitamin D supplementation have not demonstrated measurable benefits in reducing the risk of stroke (15, 26–28). Therefore, we performed a meta-analysis of published RCTs to investigate whether vitamin D supplementation could prevent stroke. Previous meta-analyses reported negative results, but none carried out targeted subgroup analysis, which was done in our study to answer questions concerning specific patients, types of interventions, and specific-influencing factors. We extended the results of earlier studies and included the latest RCTs. In total, twenty-four RCTs were included in this meta-analysis with 86,202 participants, making this study the largest meta-analysis of the association of vitamin D supplementation and the risk of stroke to be conducted thus far.

## Materials and methods

### Literature retrieval strategy

Our meta-analysis was performed in accordance with the Preferred Reporting Items for Systematic Reviews and Meta-Analyses (PRISMA) statement (29). To explore the association between vitamin D supplementation and the risk of stroke, a search was conducted to identify RCTs that assessed the association of vitamin D supplementation and stroke. A literature search strategy was designed that used and following Medical Search Terms (MeSH) from the United States National Library of Medicine: “Vitamin D,” “Calcitriol,” “Calcifediol,” “Cholecalciferol,” “25-Hydroxyvitamin D 2,” “Ergocalciferols,” “Stroke,” and stroke-derived terms [cerebrovascular accident, CVA, cerebrovascular apoplexy, apoplexy, brain vascular accident, cerebrovascular stroke, cerebral stroke]. We searched articles published before January 2022 in the following databases: PubMed, Web of Science, EMBASE, and The Cochrane Library. We also examined the references found in published meta-analyses for additional RCTs and searched the http://www.ClinicalTrials.gov website for information on registered RCTs. In total, two investigators (JF and JS) carried out the article retrieval independently. Any inconsistencies that arose were resolved by the third investigator (CZ).

### Study selection

The two investigators read the titles and abstracts of all articles independently (JF and CZ). All the RCTs that focused on vitamin D supplementation were considered for inclusion. The following types of studies were excluded: reviews, meta-analyses, conference abstracts, animal experiments, and case studies. So far more than 3,000 synthetic vitamin D compounds have been developed to improve the biological properties of the natural compound. However, only a few vitamin D compounds are available commercially [vitamin D3 (calciferol), vitamin D2 (ergocalciferol), calcidiol, calcitriol, calcipotriol, alfacalcidol, tacalcitol, paricalcitol, oxacalcitriol, falecalcitriol, and eldecalcitol] (30). Therefore, only RCTs that compared commercially available vitamin D preparations which have reached the market to placebos were included. If the endpoints of stroke were reported, the trial also was considered for inclusion. Whenever inconsistencies in the study selection emerged, the articles were decided by a third author (JS).

### Data extraction

In total, two investigators (JF and JS) independently extracted and entered the following data into an excel spreadsheet (Microsoft Excel 2019): authors, year of publication, study period, location, participant characteristics (age, sex, baseline 25(OH)D level, and BMI), vitamin D type and dosage, placebo, calcium, other interventions, the follow-up period, and the primary outcome. The data were double-checked and confirmed by the third investigator (CZ). The corresponding authors of studies with uncertain data or did not publish data on stroke were contacted by email, and the missing data were confirmed.

### Evaluation of literature quality

The Cochrane risk of bias tool was used independently by two investigators (JS and CZ) to assess the risk of bias within the studies (31). Discrepancies were resolved by the third author (JF). Review Manager 5.3 software was used to evaluate the literature quality evaluation and risk of bias graph creation. Details are shown in Supplementary Figures 1, 2.

### Statistical analysis

We computed pooled risk ratios (RRs) and 95% confidence intervals (95% CIs) to assess the effect of vitamin D supplementation on the risk of stroke. The *Q* statistic was used to indicate the presence or absence of heterogeneity, and the *I*^2^ index was used to quantify the degree of heterogeneity among studies. Fixed-effects models were used as pooling methods when the heterogeneity was low (*I*^2^ < 50%, *P* > 0.1 for the *Q* statistic). On the other hand, random-effects models were used when the heterogeneity was high (*I*^2^ ≥ 50%, *P* ≤ 0.1 for the *Q* statistic). Multiple subgroup analyses were performed at the beginning of the trial based on the different baseline characteristics of the participants. In total, nine subgroups were identified, namely, sex, age, follow-up period, BMI, baseline 25(OH)D level, the designated endpoint, latitude, type and dose of vitamin D, and whether or not calcium was provided. Funnel plots were used to assess the publication bias visually. Publication bias was further evaluated using the Begg's and Egger's tests, for which a *P-*value >0.05 indicated no publication bias. If publication bias was present, the trim and fill method was used to adjust the publication bias and further assess the stability of the results. The STATA16.0 software was used for statistical analysis, and Review Manager 5.3 software was used to create the forest plots.

## Results

### The literature search results

**Figure 1:**
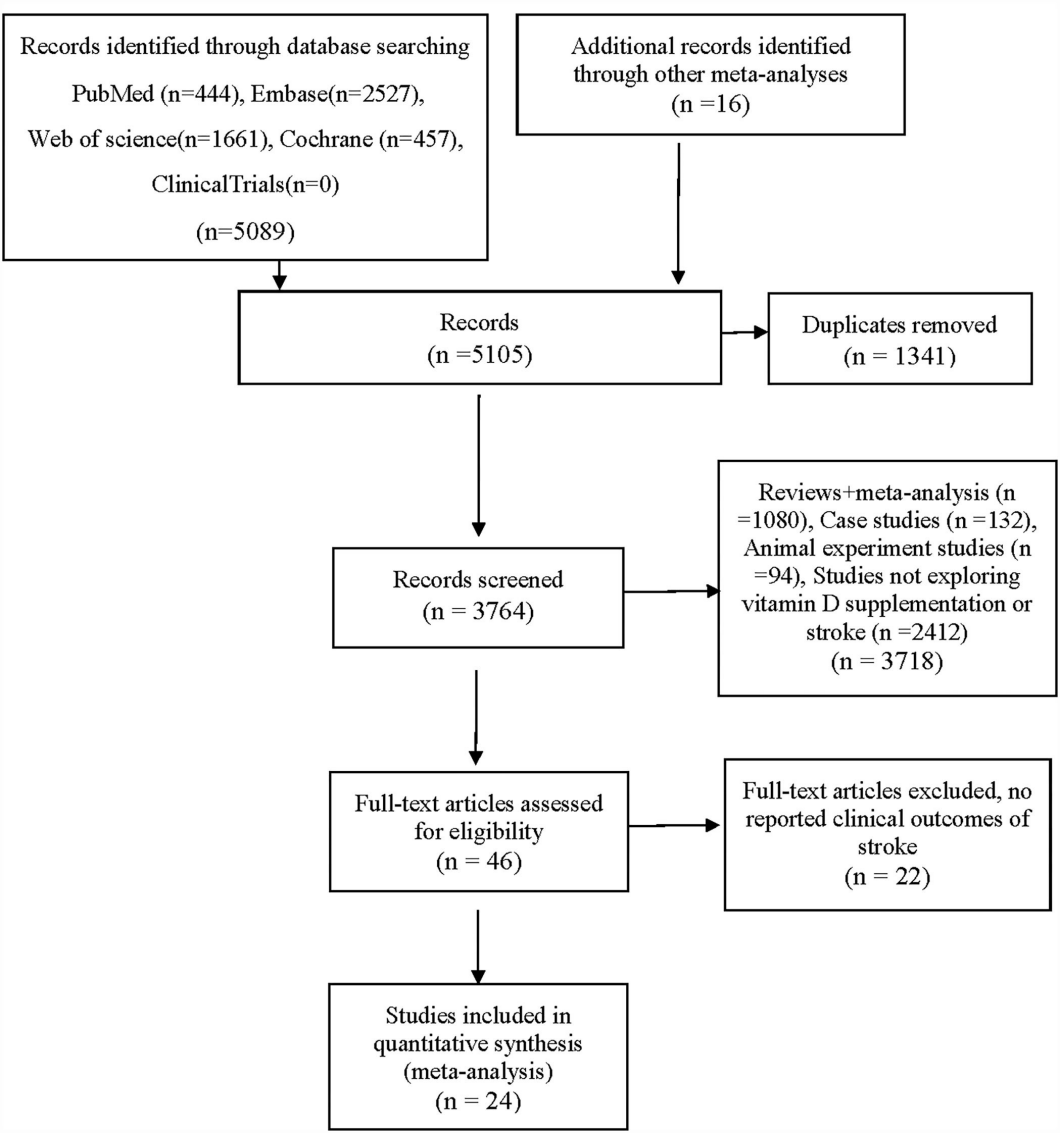
PRISMA flow chart.

After reviewing 5,089 studies obtained from the databases and 16 additional records identified through other meta-analyses, 5,081 studies were excluded, and 24 RCTs were included in the final analysis (22–25, 27, 32–50). Figure 1 shows the selection procedures and results. In total, twenty-two studies reported a definitive stroke outcome in published reports (22–25, 27, 32, 34–42, 44–50). The authors of two studies provided supplementary data on stroke (33, 43).

### Characteristics of included studies

In total, twenty-four RCTs were included in this meta-analysis, namely, 86,202 patients (22–25, 27, 32–50), of whom 43,286 received vitamin D and 42,916 received placebos with a mean follow-up of 2.85 years (range from 20 weeks to 7 years). Among participants randomized to receive vitamin D, 828/43,286 (1.91%) experienced a stroke, and 801/42,916 (1.87%) in the control group experienced a stroke. The mean age of the participants was 66.01 ± 8.51 years. Most participants (76%) were elderly women. In total, ten studies included only women, and fourteen included women and men. In total, there were 65,771 women, and 20,431 men in our meta-analysis. The primary endpoints for ten of the studies were fractures or osteoporosis. Only four trials had included stroke as a primary endpoint. Most studies (71%) administered vitamin D3, and most studies (75%) administered vitamin D doses greater than or equal to 800 IU per day. Vitamin D3 (cholecalciferol and calcitriol) was used in 17 studies with doses that ranged from 300 to 4,800 IU/d; vitamin D2 (ergocalciferol) was used in three studies, and doses ranged from 1,000 IU/d to 200,000 IU/10 weeks; in three studies, a vitamin D analog (ED-71/(1α,25-DIHYDROXY-2β-(3-hydroxypropoxy)vitaminD3, paricalcitol or alfacalcidol) was used; in one study, vitamin D dosage forms were not restricted. The baseline 25(OH)D was recorded in 14 studies and ranged from 18 to 86 nmol/L. The characteristics of the included studies are shown in Supplementary Tables 1, 2.

### Meta-analysis results

**Figure 2:**
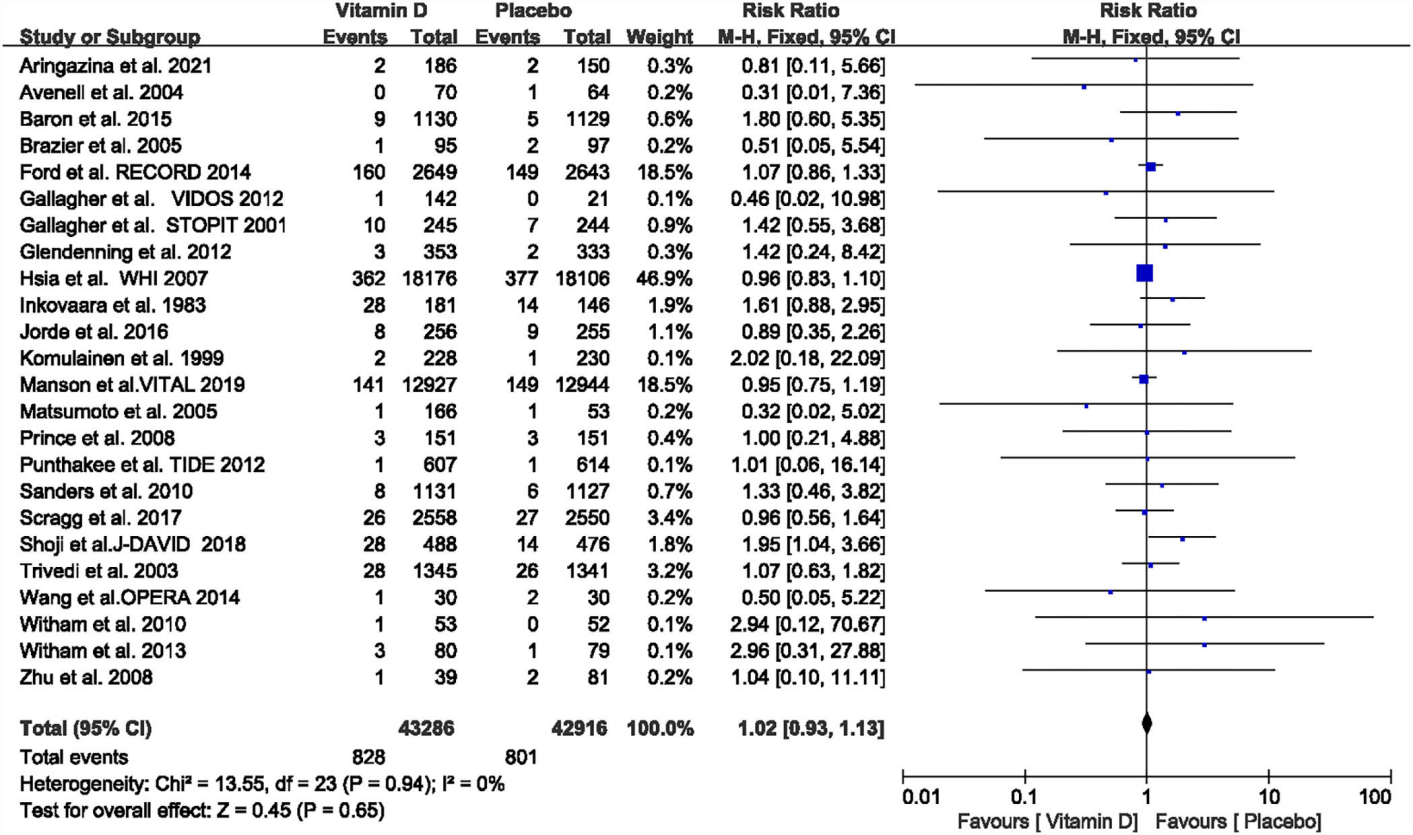
Forest plots of the association between vitamin D supplementation and risk of stroke. CI, confidence interval.

A fixed-effect meta-analysis indicated that vitamin D supplementation compared with the placebo was not associated with a reduced incidence of stroke (RR = 1.02, 95% CI: 0.93–1.13, *P* = 0.65) (Figure 2). There was no significant heterogeneity in the 24 included studies (*I*^2^ = 0.0%, *P* = 0.94). Funnel plot analysis showed no asymmetry (Supplementary Figure 3). The Begg's test (*P* = 0.44) and the Egger's test (*P* = 0.27) detected no significant small-study effects. There was no statistical heterogeneity in the subgroup analyses of the assessed participant characteristics (age, sex, BMI, follow-up period, baseline 25(OH)D level, the designated endpoint, latitude, vitamin D dosage, type, and absence or presence of concurrent calcium supplementation). The results of the subgroup analyses are shown in Table 1. The forest plots of the subgroup analyses are shown in Supplementary Figures 4–25. Tests of the bias showed no significant publication bias in the subgroup analyses except for minor publication bias in the group with a follow-up time of fewer than 3 years [the Egger's test (*P* = 0.04) and the Begg's test (*P* = 0.15)]. There were 12 studies included in the group with <3 years of follow-up time. The trim and fill method were used to adjust the publication bias in the group with follow-up times that were <3 years. The effect remained statistically insignificant (RR = 1.462, 95% CI: 0.97–2.20, *P* = 0.069) after adding imputed missing in four studies, so the meta-analysis results of groups with a follow-up time of fewer than 3 years were robust in the sensitivity analyses (the funnel plot for the trim and fill method is shown in Supplementary Figure 26). We performed a subgroup analysis for relative vitamin D supplementation doses. The following supplemental doses were considered “adequate” according to vitamin D dosages and baseline vitamin D levels: for individuals whose 25(OH)D was 50–75 nmol/L, treatment was 800 IU/d or more, and for 25(OH)D levels of 30–50 nmol/L, treatment was 1,000 IU/d or more (51). The fixed-effect meta-analysis indicated that adequate vitamin D supplementation compared with the placebo was not associated with a reduced incidence of stroke (RR = 1.13, 95% CI: 0.79–1.61, *P* = 0.51) (Supplementary Figure 27). The Begg's test (*P* = 0.21) and the Egger's test (*P* = 0.12) detected no significant small-study effects. In summary, we performed a meta-analysis on the effect of vitamin D supplementation on the risk of stroke involving 24 RCTs and 86,202 patients and found no significant benefits.

**Table 1:** Subgroup analysis of the effect of vitamin D on stroke.

**Subgroup title**	**Subgroup**	**Trials,** **no**.	**Participants, no**.	**Risk ratio**	**95% CI**	**P for interaction**	***I*^2^ (%)**	**Begg's test *P***	**Egger's test *P***
Sex	Male and female	14	42,993	1.063	0.929–1.215	0.374	0.0	0.584	0.796
	Female	10	43,209	0.983	0.857–1.127	0.803	0.0	0.592	0.195
Age(years)	≥70	12	12,750	1.134	0.949–1.356	0.167	0.0	0.837	0.468
	<70	12	73,452	0.982	0.876–1.100	0.750	0.0	0.837	0.695
Follow–up(years)	≥3	12	80,376	1.009	0.915–1.114	0.851	0.0	0.304	0.096
	<3	12	5,826	1.290	0.851–1.956	0.229	0.0	0.15	0.043
Baseline mean 25(OH)D (nmol/L)	<50	5	1,035	0.941	0.368–2.406	0.899	0.0	0.462	0.450
	≥50	9	11,872	1.138	0.807–1.604	0.462	0.0	0.348	0.226
Type of vitamin D	VitaminD3	17	83,211	1.007	0.913–1.110	0.887	0.0	0.837	0.202
	VitaminD2	3	527	1.215	0.368–4.011	0.750	0.0	0.296	0.362
Daily dose equivalent (IU)	≥1000	14	38,653	1.051	0.879–1.257	0.587	0.0	0.661	0.071
	<1000	6	45,044	0.991	0.883–1.113	0.881	0.0	1.000	0.873
Daily dose equivalent (IU)	≥2000	4	31,826	0.945	0.770–1.159	0.585	0.0	0.308	0.207
	<2000	16	51,831	1.029	0.921–1.149	0.611	0.0	0.558	0.055
Intervention	Vitamin D +calcium	6	37,593	0.971	0.846–1.116	0.681	0.0	0.707	0.466
	Vitamin D	16	41,070	1.075	0.910–1.270	0.395	0.0	0.753	0.432
BMI(kg/m^2^)	≥30	3	1,895	0.863	0.368–2.025	0.735	0.0	0.296	0.644
	<30	17	76,296	0.998	0.894–1.114	0.965	0.0	1.000	0.163
Stroke as the primary outcome	Yes	4	32,279	1.022	0.839–1.245	0.832	34.6	0.734	0.601
	No	20	53,923	1.023	0.917–1.141	0.689	0.0	0.256	0.367
Latitude	≥40°	12	10,852	1.116	0.933–1.334	0.229	0.0	0.631	0.846
	<40°	6	8,534	1.019	0.666–1.561	0.930	0.0	1.000	0.957

## Discussion

This meta-analysis included 86,202 participants from 24 RCTs. No significant correlation was observed between vitamin D supplementation and the incidence of stroke (RR = 1.02, 95% CI: 0.93–1.13, *P* = 0.65) when randomized for treatment with different doses of vitamin D supplementation. Although worldwide consensus on the optimal vitamin D status has not been ascertained, optimal 25(OH)D serum levels are generally at least 75 nmol/L (30 ng/ml), with levels of 50–75 nmol/L (20–30 ng/ml) considered “insufficient,” and levels below 50 nmol/L (20 ng/ml) considered “deficient” (52). Baseline 25(OH)D levels were provided in 15 of our included studies, 14 of which were eligible for meta-analysis. The baseline 25(OH)D levels in 12 studies were insufficient or deficient. The administered daily dose equivalent of vitamin D ranged from 300 to 3,225 IU in the included studies, and in most studies (14 of 20) was ≥ 1,000 IU. For individuals whose 25(OH)D is 50–75 nmol/L, treatment with 600–800 IU/d of vitamin D3 is usually sufficient, and for 25(OH)D levels of 30–50 nmol/L, treatment usually includes 800 to 1,000 IU/d or more (51). Therefore, we believe that the vitamin D dose provided in most studies was adequate. We conducted a subgroup analysis of baseline 25(OH)D levels with 50 nmol/L as the division between groups and a daily dose equivalent of vitamin D at 1,000 IU and 2,000 IU as the division. However, no meaningful difference emerged in the subgroup analysis. In addition, no significant differences were observed in the subgroup analyses based on age, sex, BMI, follow-up time, the designated endpoint, latitude, vitamin D type, and absence or presence of concurrent calcium supplementation.

According to recent meta-analyses involving 15 RCTs and more than 80,000 participants (15), there was no significant benefit from vitamin D supplementation for stroke prevention (Su et al.: HR = 1.05, 95% CI: 0.96–1.14, *P* = 0.425). In total three other meta-analyses (26, 28, 53) also failed to demonstrate a reduction in stroke incidence with vitamin D supplementation. All the four meta-analyses included more than ten studies, and the search strategies revolved around cardiovascular events, stroke, and vascular outcomes. Our study complemented some of the limitations of the aforementioned studies. We performed a subgroup analysis to assess the influence of latitude on the effect of vitamin D supplementation and compared the risk of stroke in patients with different vitamin D levels and different supplementation dosages. We also evaluated the effect of relative vitamin D supplementation doses (according to the baseline 25(OH)D levels vs. vitamin D supplementation doses) on the incidence of stroke, which better reflects the benefit of supplementation. Our study performed subgroup analyses of the participant characteristics, intervention form, and environmental effects, and the results were generally consistent with the studies aforementioned. In conclusion, despite differences in the number and criteria inclusion, current meta-analyses consistently demonstrate no significant benefit of vitamin D supplementation in stroke prevention.

Even though existing studies have shown that vitamin D supplementation is ineffective in preventing stroke occurrence, several meta-analyses have indicated that low-serum 25(OH)D levels were related to an increased risk of stroke (13–15). In addition, an increasing risk of symptomatic ischemic stroke has been associated with decreasing plasma 25(OH)D levels (13). The contradictory results of these studies are challenging to explain. This paradoxical result implied that the relationship between low-serum 25(OH)D levels and stroke occurrence might not be causal.

Due to the observational nature of this meta-analysis, several confounding variables might have affected the final results. First, only a few trials included the endpoint of stroke as a primary focus. Data for these secondary endpoints may not be collected or accepted for review in the same way as data for the primary endpoint in the trials. However, this possibility was unlikely to introduce differential bias between the groups. Most trials of vitamin D supplementation included fractures or osteoporosis as the primary endpoint, and the study populations primarily included older patients and postmenopausal women. Therefore, there was wide variability in the baseline patient characteristics in the included RCTs. For this reason, we conducted a subgroup analysis of whether stroke was included in the primary endpoint. The results indicated no significant difference in outcome regardless of whether stroke was the primary endpoint. This result demonstrated that the endpoint assessed might not affect the outcome. The ratio of men to women was approximately 1:3 in our study. We performed a subgroup analysis based on sex. In total, fourteen studies included women and men, with 22,562 women, and 20,431 men in this subgroup. After subgroup analysis, the effect remained statistically insignificant (ReffectivR = 1.063, 95% CI: 0.929–1.215, *P* = 0.374). Second, factors such as obesity (54), advanced age, and malnutrition caused by co-morbid chronic diseases (28) could lead to differences in the effectiveness of individual supplementation at equivalent supplementation doses. However, these differences seemed unlikely to affect the final results due to the increases in 25(OH)D levels after vitamin D supplementation in most studies. Because of the characteristics of vitamin D metabolism, the primary source of vitamin D is skin production induced by sunlight [Ultraviolet-B (UV)-B], and nutrient intake usually accounts for only a small proportion (55). Thus, the degree of outdoor activity of participants would directly affect the level of vitamin D they produce. Notably, sun exposure, as influenced by seasons, geography, outdoor activities, and others, has rarely been studied with respect to the incidence of stroke (17). Additional studies of stroke risk and 25(OH)D levels associated with relative sun exposure are needed.

Previous observational studies have supported the hypothesis that vitamin D supplementation could result in a decreased occurrence of stroke. However, most clinical trials have been negative. A previous meta-analysis suggested that it was unrealistic to prescribe vitamin D supplements to prevent stroke, cardiovascular disease, cancer, and even fractures, and it might be futile to conduct similar vitamin D trials to investigate these endpoints (56). We agree based on our data. We determined that the randomized controlled trials done so far have provided sufficient evidence, and further studies of similar design to the existing trials are unlikely to alter the results. Therefore, large RCTs with several outcomes intrinsically different from the studies included herein are needed to provide convincing evidence that any small treatment effect is a meaningful discovery. The consistency of results across studies completed to date suggests that the likelihood of reporting such results is low.

## Limitations

There are several limitations associated with this study: a majority of the trials were small and did not pre-designate stroke as the primary endpoint. The baseline characteristics of the included RCTs varied widely, such as the population covered by the study, follow-up period, form, dose, frequency of vitamin D supplementation, and others. Subgroup analysis mitigated these differences to some extent but also had the disadvantage of reducing the sample size, which diminished the strength of the final evidence. The use of low-dose vitamin D supplementation in the control group would also weaken the power of evidence. However, there was only one study with this problem, and because of the small sample size, we do not believe that this had a critical impact on the overall results. In addition, due to a lack of patient-level data, some subgroups such as sun exposure and eating habits could not be examined.

## Conclusion

In conclusion, our meta-analysis suggested that extra supplementation of vitamin D did not provide any benefit in decreasing the occurrence of stroke. Therefore, our hypothesis that the risk of stroke would be reduced with vitamin D supplementation was not validated. Because numerous studies have already reached the same conclusion, we believe that it is unnecessary to conduct additional similar experiments to assess whether vitamin D supplementation reduces the risk of stroke. Notably, the problems of stroke risk and 25(OH)D levels associated with relative sun exposure should be addressed in future trials.

## Data availability statement

The original contributions presented in the study are included in the article/Supplementary material, further inquiries can be directed to the corresponding author/s.

## Author contributions

JF contributed to the conceptualization, manuscript compilation, data collection, and final approval. JS and CZ were responsible for the manuscript compilation and data collection. All authors contributed to the article and approved the submitted version.

## Conflict of interest

The authors declare that the research was conducted in the absence of any commercial or financial relationships that could be construed as a potential conflict of interest.

## Publisher's note

All claims expressed in this article are solely those of the authors and do not necessarily represent those of their affiliated organizations, or those of the publisher, the editors and the reviewers. Any product that may be evaluated in this article, or claim that may be made by its manufacturer, is not guaranteed or endorsed by the publisher.
