# Supplementary material for: Vitamin D supplementation and risk of stroke: A meta-analysis of randomized controlled trials
Source: Front Neurol. 2022 Aug 18;13:970111. doi: 10.3389/fneur.2022.970111 (PMC9434369; doi:10.3389/fneur.2022.970111)
Supplement: Supplementary file 1 [file Data_Sheet_1.docx]

Supplementary Material

# Supplementary Figures


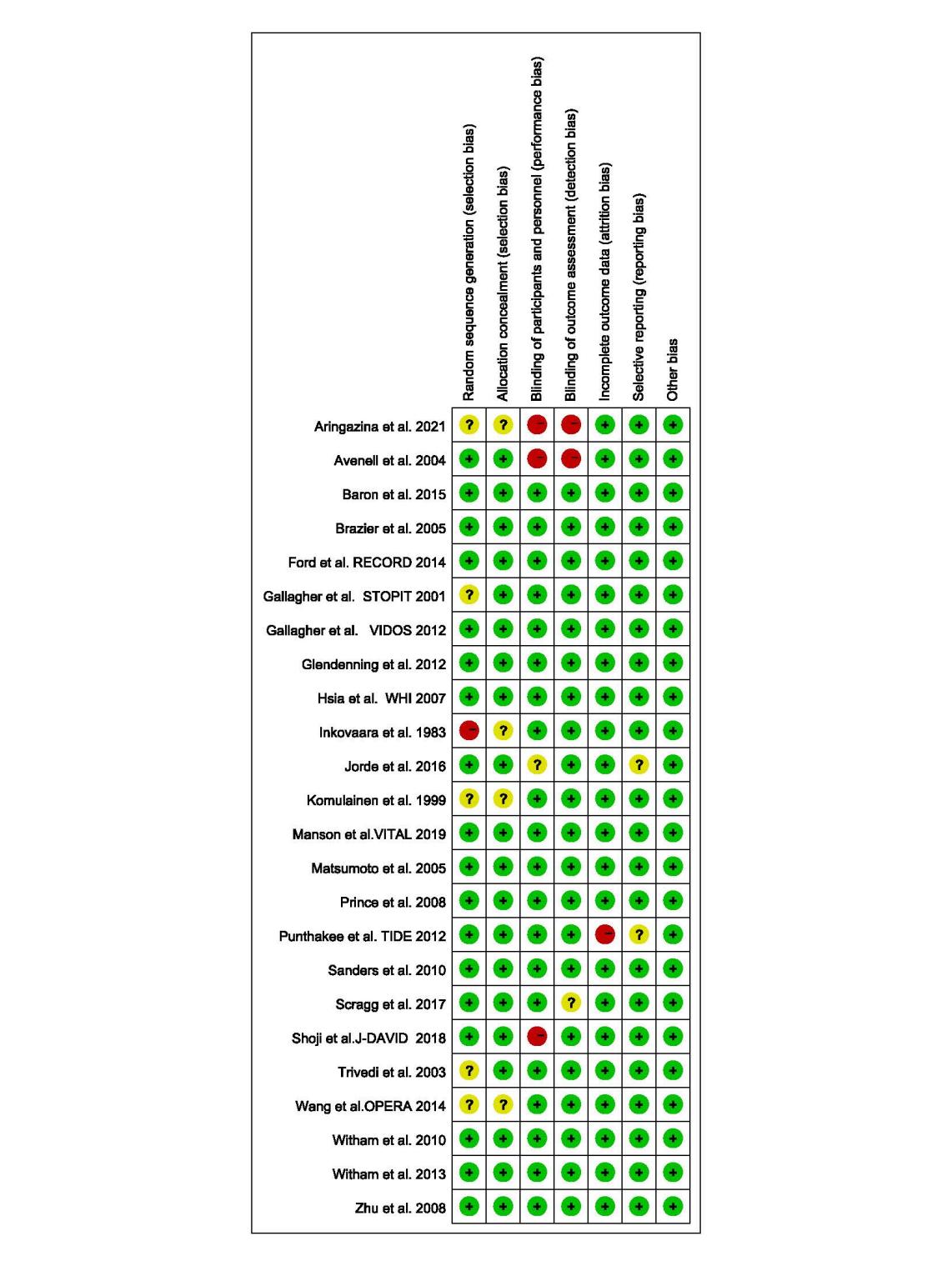


**Supplementary Figure 1.** Risk of bias graph


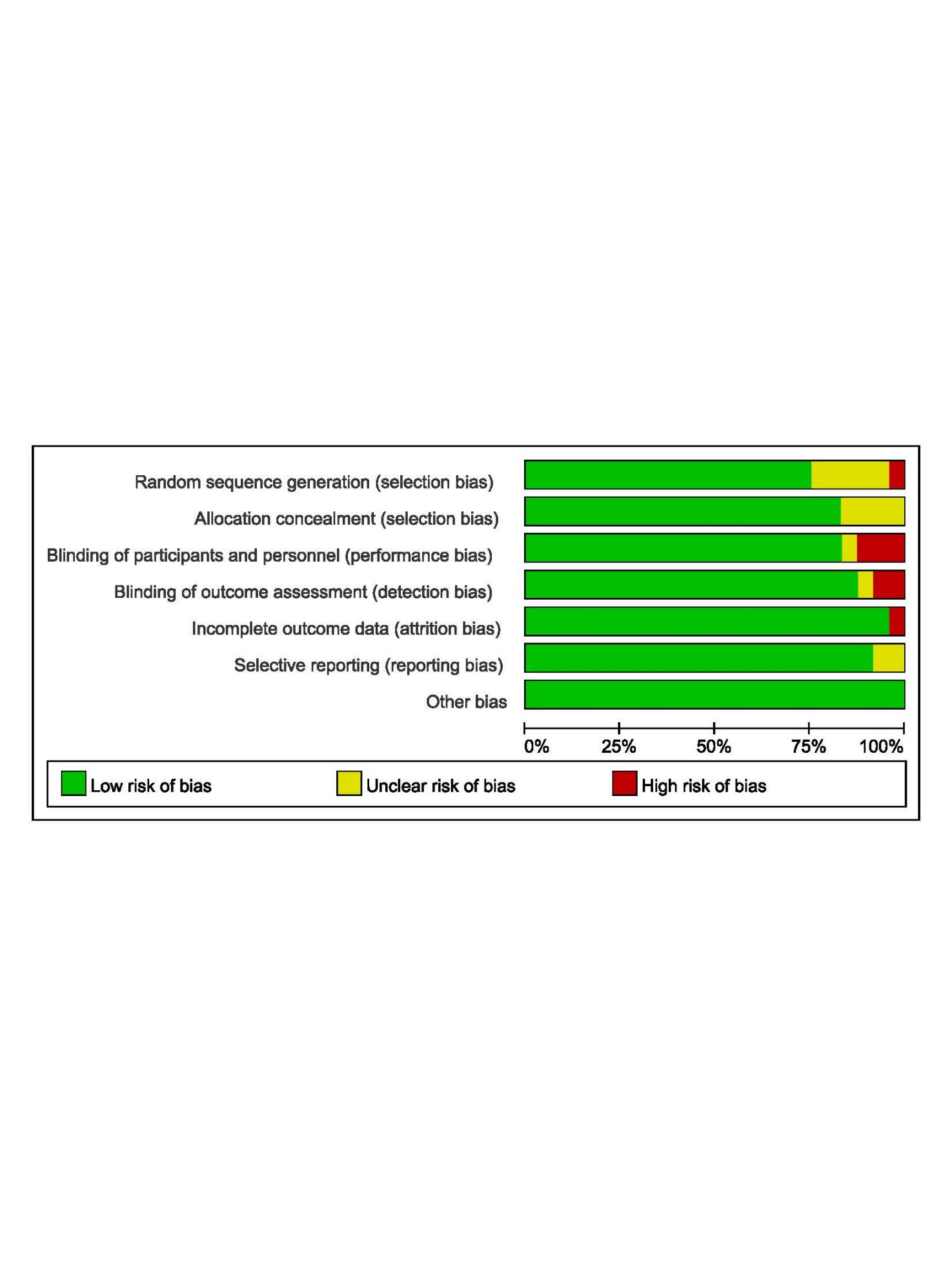


**Supplementary Figure 2.** Risk of bias summary

**
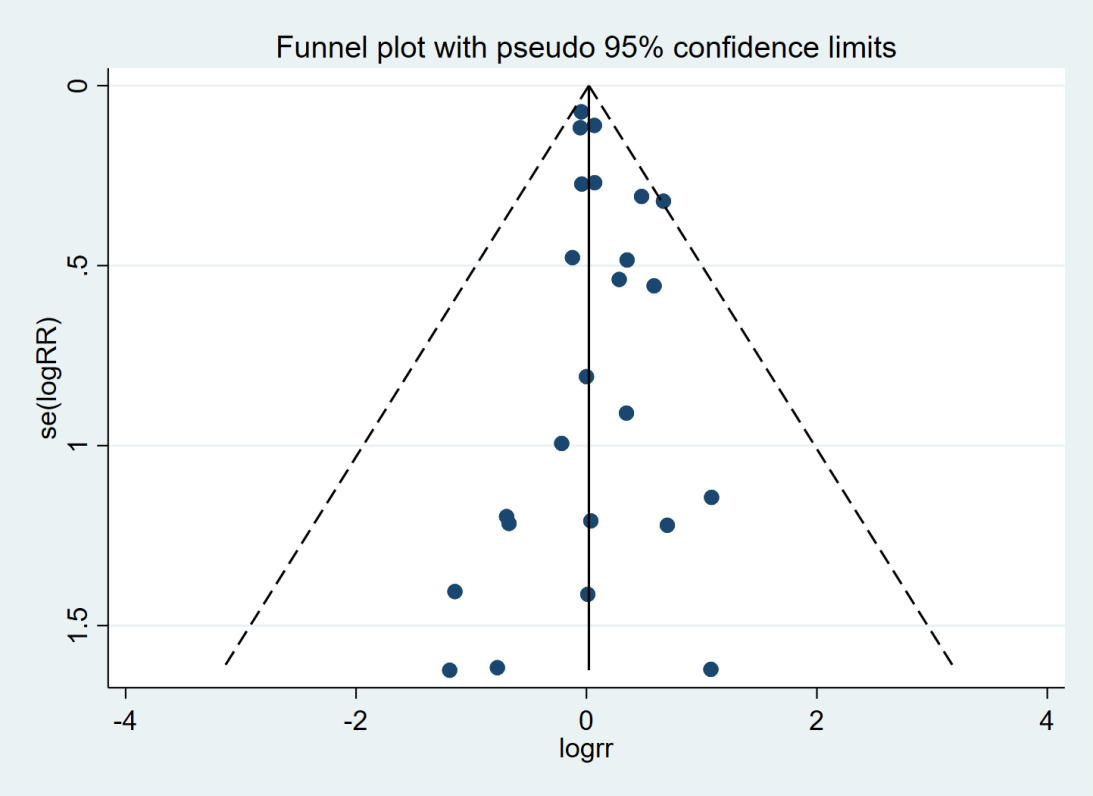
**

**Supplemental figure 3.** Funnel Plot for Stroke.

**
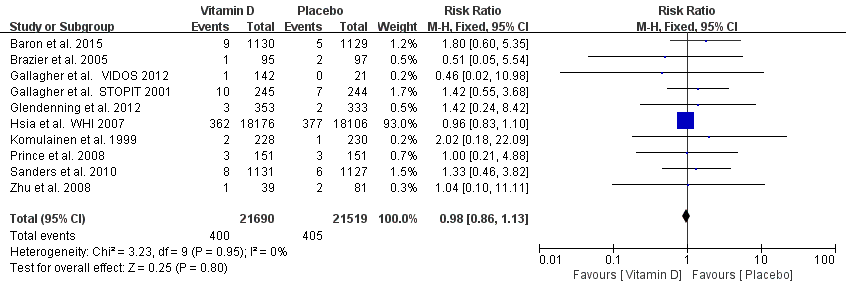
**

**Supplemental figure 4.** The forest plot of sex of female group


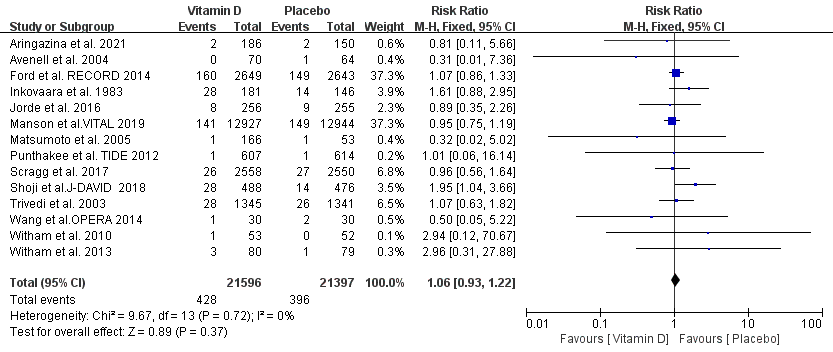


**Supplemental figure 5.** The forest plot of sex of male and female group


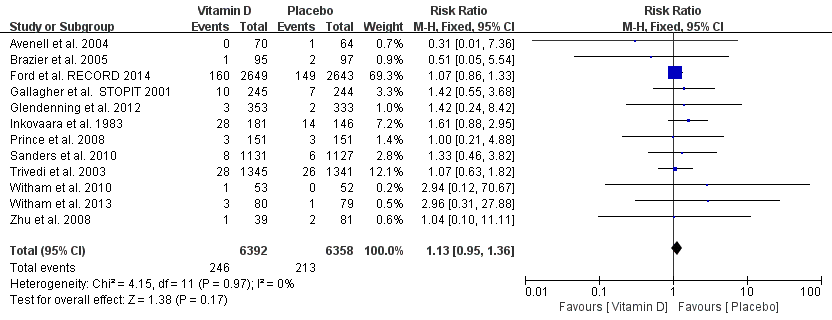


**Supplemental figure 6.** The forest plot of age ≥70 group


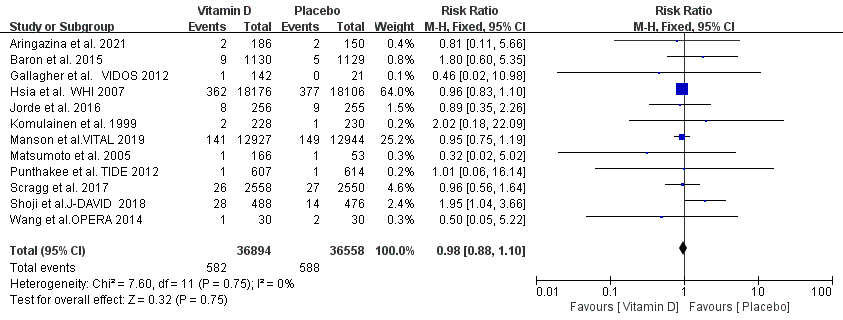


**Supplemental figure 7.** The forest plot of age <70 group


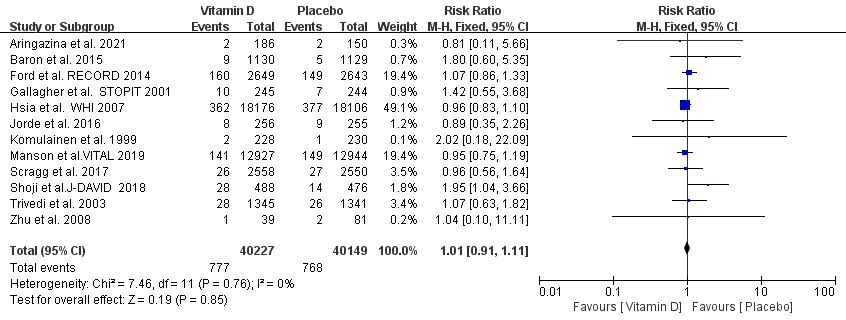


**Supplemental figure 8.** The forest plot of follow-up time ≥3 years group


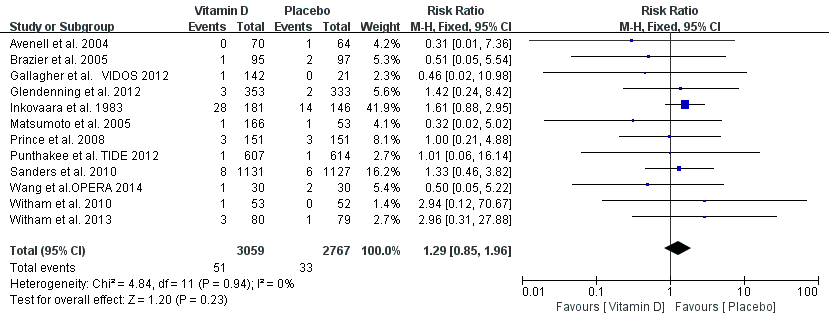


**Supplemental figure 9.** The forest plot of follow-up time <3 years group


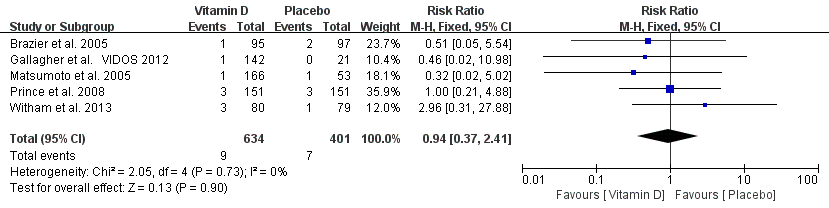


**Supplemental figure 10.** The forest plot of baseline mean 25(OH)D <50 group


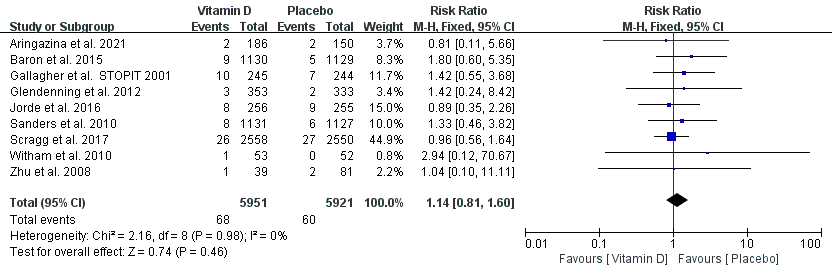


**Supplemental figure 11.** The forest plot of baseline mean 25(OH)D ≥50 group


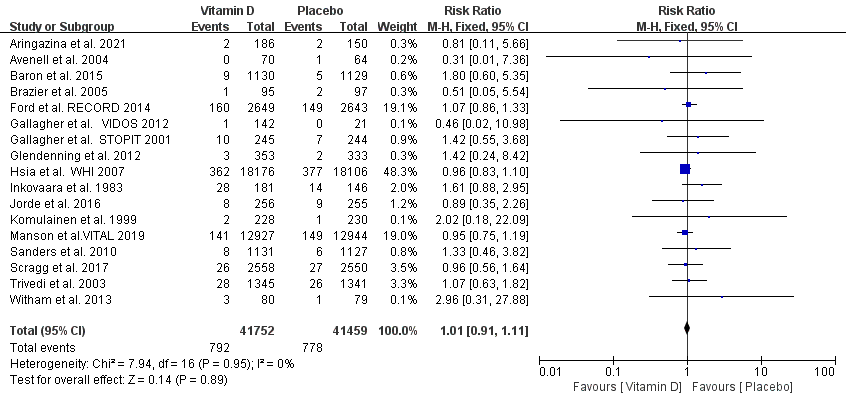


**Supplemental figure 12.** The forest plot of VitaminD3 group


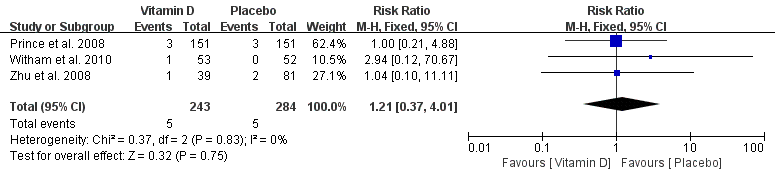


**Supplemental figure 13.** The forest plot of VitaminD2 group


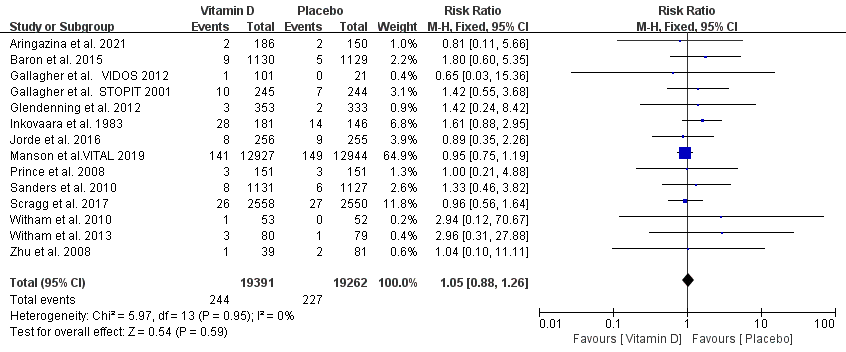


**Supplemental figure 14.** The forest plot of daily dose equivalent ≥1000 group


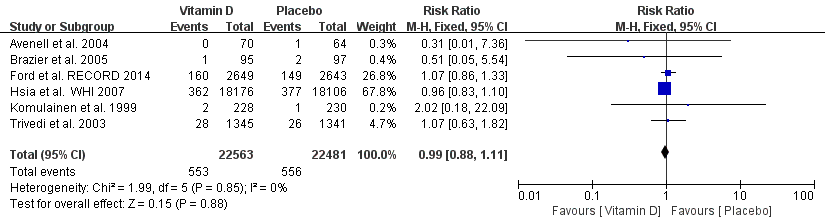


**Supplemental figure 15.** The forest plot of daily dose equivalent <1000 group


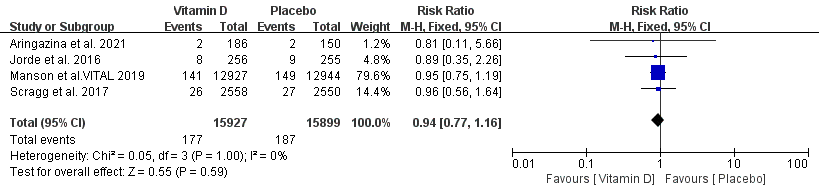


**Supplemental figure 16.** The forest plot of daily dose equivalent ≥2000 group


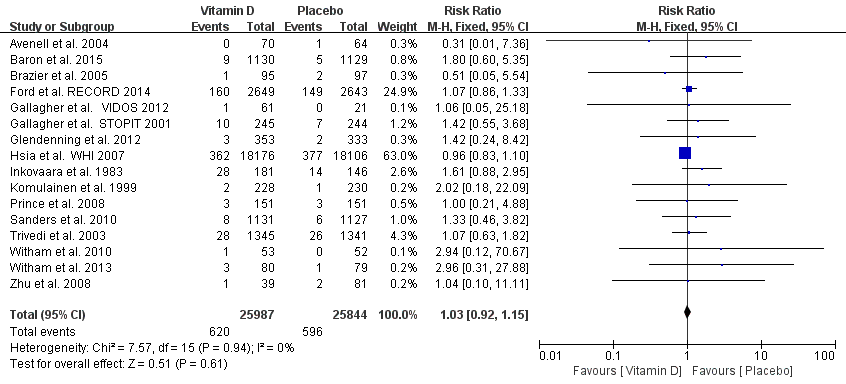


**Supplemental figure 17.** The forest plot of daily dose equivalent <2000 group


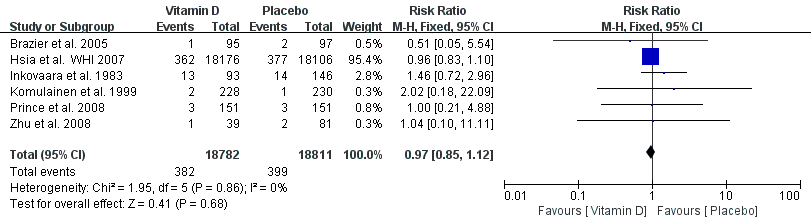


**Supplemental figure 18.** The forest plot of vitamin D + calcium group


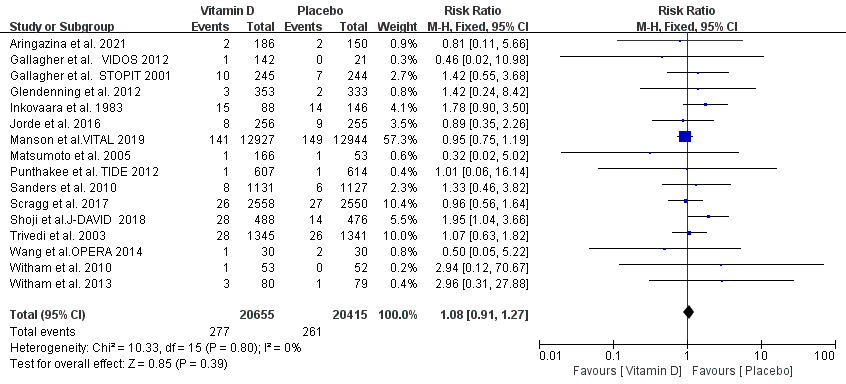


**Supplemental figure 19.** The forest plot of vitamin D without calcium group


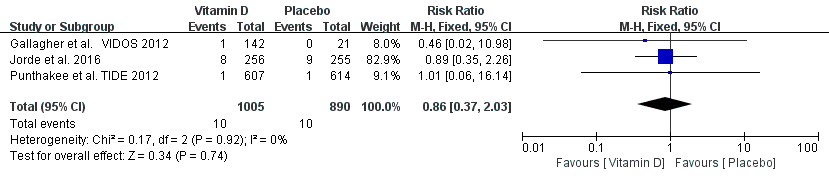


**Supplemental figure 20.** The forest plot of BMI ≥30 group


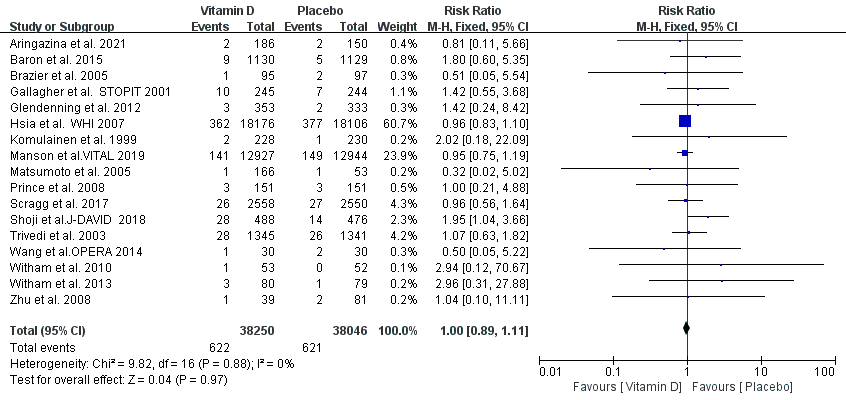


**Supplemental figure 21.** The forest plot of BMI <30 group


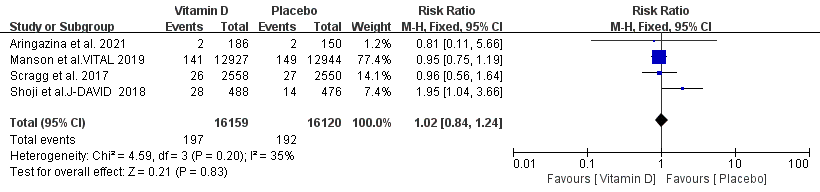


**Supplemental figure 22.** The forest plot of stroke as the primary outcome group


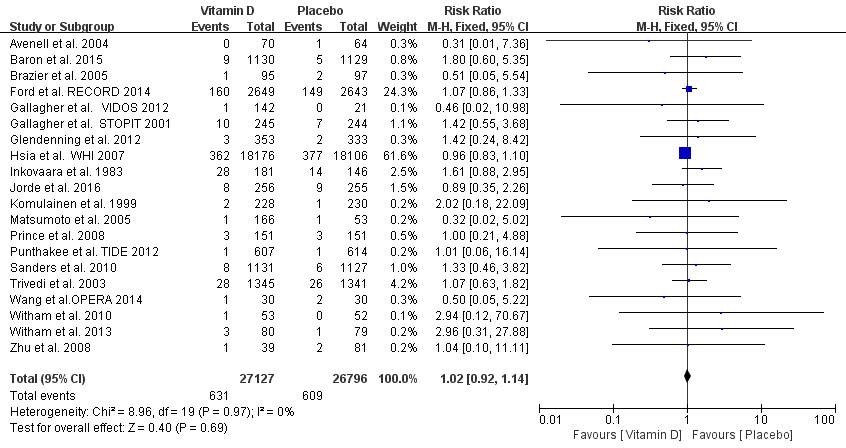


**Supplemental figure 23.** The forest plot of stroke not the primary outcome group


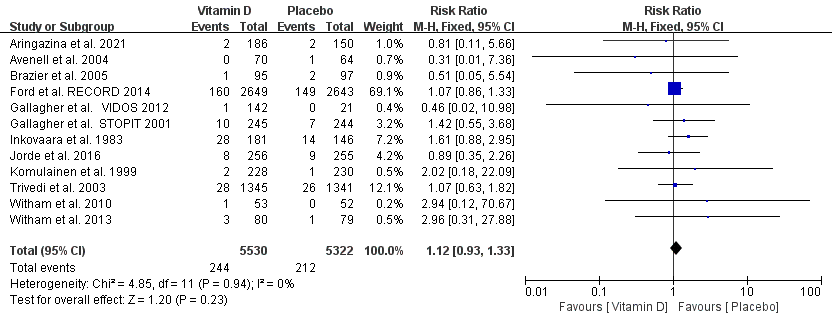


**Supplemental figure 24.** The forest plot of latitude ≥40° group


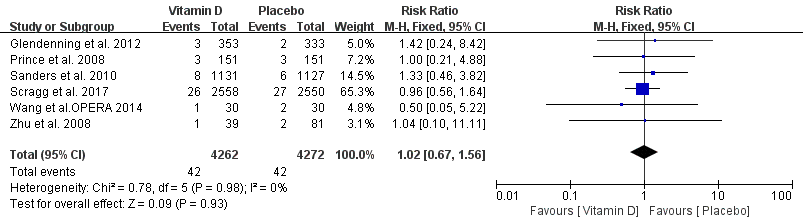


**Supplemental figure 25.** The forest plot of latitude <40° group


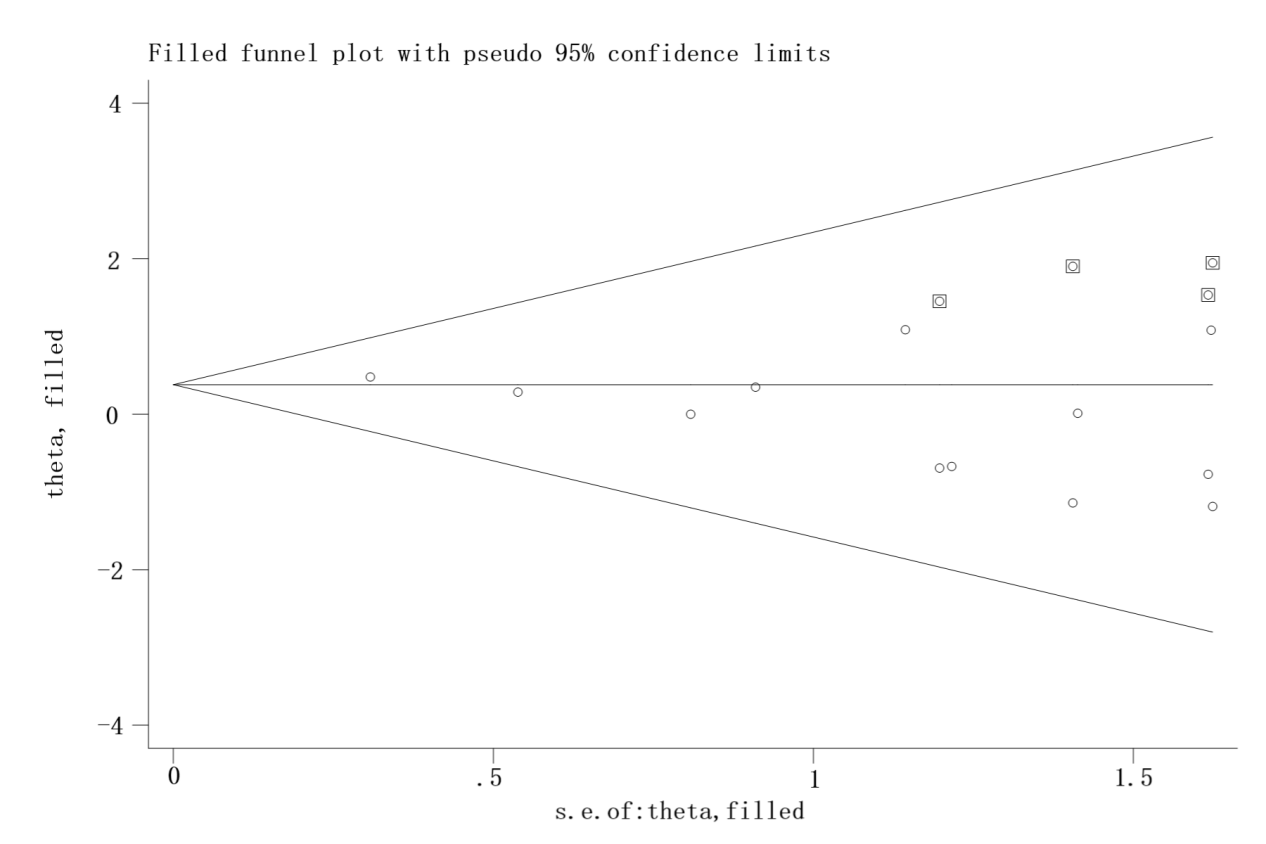


**Supplemental figure 26.** The funnel plot of trim and fill method of follow-up time <3 years group


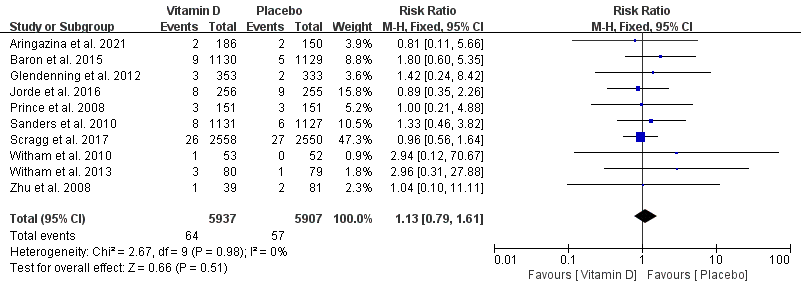


**Supplemental figure 27.** The forest plots of the association between adequate vitamin D supplementation and risk of stroke

# Supplementary Tables

**Supplementary Table 1.** Characteristics of the Involved Trials

| Source | Year of publication | Location | Follow-up (y) | Participants | Study Period | Vitamin D Type and Dosage | Calcium and other intervention | Control | Primary outcome |
| --- | --- | --- | --- | --- | --- | --- | --- | --- | --- |
| Inkovaara et al. | 1983 | Finland | 1 | residents aged 65-97 years of home for the aged | 1978 | Vitamin D3, 1000 IU/d | With or without calcium carbonate 3 g/d, methandienone 2.5 mg/d | Placebo, calcium carbonate 3 g/d, methandienone 2.5 mg/d | Bone fracture |
| Komulainen et al. | 1999 | Finland | 5 | non-osteoporotic postmenopausal women aged 47-56 years | 1990–1991 | Vitamin D3, 300 IU/d for 4 years then 100 IU/d | Calcium lactate 500 mg/d with or without HRT | Placebo + calcium lactate, HRT | BMD |
| Gallagher et al. STOPIT | 2001 | United States | 3 | women aged 65-77 years with normal bone density for their age | NA | Calcitriol, 0.25 μg twice daily | with or without HRT/ERT | Placebo, HRT/ERT | Change in BMD of the femoral neck and spine |
| Trivedi et al. | 2003 | United Kingdom | 5 | men and women aged 65-85 years living in the community | 1996-1997 | Cholecalciferol, 100 000 IU/4 months | NA | Placebo | Fracture incidence and total mortality |
| Avenell et al. | 2004 | United Kingdom | 1 | people aged ≥ 70 years who had previously sustained a fracture | 2000-2001 | Vitamin D3, 800 IU/d | With or without calcium 1000 mg /d | Placebo, calcium 1000 mg /d | Eligible participants recruited |
| Brazier et al. | 2005 | France | 1 | community-dwelling ambulatory women (aged >65 years) with vitamin D insufficiency (serum 25(OH)D≤12 ng/mL) | NA | Cholecalciferol, 400 IU twice daily | calcium carbonate 500 mg twice daily | Placebo | BMD and biochemical markers of bone formation and resorption |
| Matsumoto et al. | 2005 | Japan | 1 | osteoporotic patients. Female subjects were at least 3 years after menopause or aged >60 years | NA | 0.5, 0.75, or 1.0 mg ED-71/d | NA | Placebo | change in lumbar BMD |
| Hsia et al. WHI | 2007 | United States | 7 | Women with no evidence of a medical condition associated with anticipated survival <3 years and no safety, adherence or retention risks | 1995-2000 | Vitamin D3, 400 IU/d | calcium carbonate 1000mg/d | Placebo | Bone fracture |
| Prince et al. | 2008 | Australia | 1 | community-dwelling ambulant older women aged 70-90 years, with a serum 25(OH)D≤24.0 ng/mL and a history of falling in the previous year | 2003-2004 | ergocalciferol, 1000 IU/d | Calciuma 1000 mg/d | Placebo +calcium 1000 mg/d | Number of falls |
| Zhu et al. | 2008 | Australia | 5 | ambulatory women aged 70-80 years | 1998 | ergocalciferol, 1000 IU/d | 1200mg/d calcium | Placebo, calcium 1200 mg/d | hip BMD |
| Witham et al. | 2010 | United Kingdom | 0.38 | patients with systolic heart failure aged ≥70 years with 25(OH)D levels< 50 nmol/L | 2005-2008 | 200 000 U of oral vitaminD2/10weeks | NA | Placebo | 6 min walk test |
| Sanders et al. | 2010 | Australia | 2.96 | Community-dwelling women aged≥70 years at high risk of fracture | 2003-2005 | Vitamin D3, 500 000 IU/y | NA | Placebo | falls and fractures |
| Gallagher et al. VIDOS | 2012 | United States | 1 | healthy, white, postmenopausal women with vitamin D insufficiency (25(OH)D <50 nmol/L) | 2007-2008 | Vitamin D3: 400, 800, 1600, 2400, 3200, 4000 and 4800 IU/d | NA | Placebo | 25(OH)D and PTH |
| Punthakee et al. TIDE | 2012 | 33 countries | 0.44 | people with type 2 diabetes and other cardiovascular risk factors aged ≥50 years | 2009-2010 | Vitamin D 1000 IU/d | NA | Placebo | cancer or all-cause death |
| Glendenning et al. | 2012 | Australia | 0.75 | community-dwelling ambulant women aged ≥70 years | 2009.2-2009.7 | Cholecalciferol 150 000 IU/3months | NA | Placebo | Falls, Mobility and Muscle Strength |
| Witham et al. | 2013 | United Kingdom | 1 | Patients ≥70 years with isolated systolic hypertension (supine systolic BP>140 mmHg; supine diastolic BP<90 mmHg) and baseline 25(OH)D <30 ng/mL | 2009-2001 | Vitamin D3, 100 000 IU/3months | NA | Placebo | change in BP, arterial stiffness, endothelial function, cholesterol level, insulin resistance and B-type natriuretic peptide level |
| Wang et al. OPERA | 2014 | CHINA Hong Kong | 1 | Patients with stages 3-5 chronic kidney disease and left ventricle hypertrophy | 2008-2010 | Paricalcitol, 1μg/d | NA | Placebo | change in left ventricle mass index measured by cardiac magnetic resonance imaging |
| Ford et al. RECORD | 2014 | United Kingdom | 6.2 | Participants aged ≥70 y who had had a low trauma, osteoporotic fracture in the previous 10 y | 1999-2002 | Vitamin D3, 800 IU/d | With or without calcium 1000 mg /d | Placebo, calcium 1000 mg /d | new low-energy fractures |
| Baron et al. | 2015 | United States | 3 | patients with recently diagnosed adenomas and no known colorectal polyps remaining after complete colonoscopy | 2004-2008 | Vitamin D3, 1000 IU/d | With or without calcium carbonate 1200 mg/d | Placebo with or without calcium carbonate 1200 mg /d | colonic adenoma |
| Jorde et al. | 2016 | Norway | 5 | patients with prediabetes | 2007-2008 | Vitamin D3, 20 000 IU/weeks | NA | Placebo | Progression to T2DM |
| Scragg et al. | 2017 | New Zealand | 3.3 | community-resident adults aged 50-84 years | 2011-2015 | Vitamin D3, Initial 200 000 IU, then 100 000 IU/months | NA | Placebo | cardiovascular disease and death |
| Shoji et al. J-DAVID | 2018 | Japan | 4 | patients aged 20-80 years receiving maintenance hemodialysis without secondary hyperpara thyroidism | 2011-2015 | Alfacalcidol, 0.5 μg/d | NA | Placebo | fatal and nonfatal cardiovascular events |
| Manson et al. VITAL | 2019 | United States | 5.3 | people had no history of cancer (except nonmelanoma skin cancer) or cardiovascular disease (men ≥50 years, women ≥55 years) | 2011-2014 | Vitamin D3, 2000 IU/d | With or without marine omega-3 fatty acids | Placebo with or without marine omega-3 fatty acids | invasive cancer of any type and major cardiovascular events |
| Aringazina et al. | 2021 | Kazakhstan | 4 | outpatients aged 50-60 years who had no cardiovascular diseases | 2012-2019 | vitamin D3, 2000 IU/d | NA | Placebo | Metabolic Syndrome and Cardiovascular Diseases |

HRT, hormone replacement therapy; BMD, bone mineral density; STOPIT, Estrogen and Calcitriol in the Prevention of Age-Related Bone Loss; NA, not available; ERT, estrogen replacement therapy; ED-71,1a,25-dihydroxy-2b-(3-hydroxypropyloxy)vitamin D3; 25(OH)D, 25-hydroxyvitamin D; WHI, Women’s Health Initiative clinical trial; VIDOS, Vitamin D Supplementation in Older Subjects; PTH, parathyroid hormone; TIDE, Thiazolidinedione Intervention with vitamin D Evaluation; BP, blood pressure; OPERA, Oral Paricalcitol in Retarding Cardiac Hypertrophy, Reducing Inflammation and Atherosclerosis in Stage 3-5 Chronic Kidney Disease; RECORD, Randomised Evaluation of Calcium Or vitamin D; J-DAVID, Japan Dialysis Active Vitamin D trial; VITAL, The Vitamin D and OmegA-3 Trial

**Supplementary T****able 2.** Patients’ Demographic and Clinical Characteristics

| Source | Patients, No. | Intervention/Control,No . | Sex  Male/female, No. | | Age  Mean ±SD, y | | BMI  Mean ±SD, kg/m^2^ | | Baseline 25(OH)D  Mean (SD) nmol/l | |
| --- | --- | --- | --- | --- | --- | --- | --- | --- | --- | --- |
|  |  |  | Vitamin D | Control | Vitamin D | Control | Vitamin D | Control | Vitamin D | Control |
| Inkovaara et al. | 327 | 181/146 | 26/155 | 31/115 | 79.5 ±7.1 | | NA | NA | NA | NA |
| Komulainen et al. | 458 | 228/230 | 0/228 | 0/230 | 53 | | 27 | | NA | NA |
| Gallagher et al. STOPIT | 489 | 245/244 | 0/245 | 0/244 | 71±3.5 | 71.4±4 | 27 | | 78.9±25.7 | 78.9±25.9 |
| Trivedi et al. | 2686 | 1345/1341 | 1019/326 | 1018/323 | 74.8±4.6 | 74.7±4.6 | 24.3±3.4 | 24.4±3.0 | NA | NA |
| Avenell et al. | 134 | 70/64 | 23/111 | | 76.7±4.7 | | NA | NA | NA | NA |
| Brazier et al. | 192 | 95/97 | 0/95 | 0/97 | 74.2±6.4 | 75.0±7.3 | 27.0±4.4 | 26.4±4.3 | medians 18.22 | medians 17.47 |
| Matsumoto et al. | 219 | 166/53 | 4/215 | | 66.9±7.3 | 68.0±7.7 | 21.8±3.1 | 22.7±2.9 | 43.0±14.3 | 43.1±14.2 |
| Hsia et al. WHI | 36282 | 18176/18106 | 0/18176 | 0/18106 | 62.4±7.0 | 62.4±6.9 | 29.1±5.9 | 29.0±5.9 | NA | NA |
| Prince et al. | 302 | 151/151 | 0/151 | 0/151 | 77.0 ±4.2 | 77.4±5.0 | 29.3 | 28.2 | 45.18±12.48 | 44.18±12.73 |
| Zhu et al. | 120 | 39/81 | 0/39 | 0/81 | 75.4±2.7 | 74.4±2.4 | 27.5 | 27.9 | 70.2±25.6 | 66.9±30.2 |
| Witham et al. | 105 | 53/52 | 34/19 | 35/17 | 78.8±5.6 | 80.6±5.7 | 27.2 ±5.1 | 27.3 ±4.5 | 51.17±22.21 | 59.16±24.96 |
| Sanders et al. | 2258 | 1131/1127 | 0/1131 | 0/1125 | Median (IQR)  76.0(73.1-80.2) | Median (IQR)  76.1(73.0-79.7) | NA | NA | median (IQR) 53 (40-65) | median (IQR)  45 (40-57) |
| Gallagher et al. VIDOS | 163 | 142/21 | 0/142 | 0/21 | 66.9±7.3 | 66±6.5 | 30.1±5.8 | 31.1±5.3 | 38.3±9.5 | 37.7±9.1 |
| Punthakee et al. TIDE | 1221 | 607/614 | 245/362 | 254/360 | 66.7±6.7 | 66.6±6.3 | 30.6±5.3 | 30.7±5.3 | NA | NA |
| Glendenning et al. | 686 | 353/333 | 0/353 | 0/333 | 76.9 ± 4.0 | 76.5 ± 4.0 | 27.5 ± 4.6 | 27.4 ±4.9 | 65.0±17.8 | 66.5±27.1 |
| Witham et al. | 159 | 80/79 | 40/40 | 42/37 | 76.9± 4.8 | 76.7 ±4.5 | 28.5 ±5.0 | 27.9±4.5 | 44.93±14.98 | 44.93±14.98 |
| Wang et al. OPERA | 60 | 30/30 | 18/12 | 14/16 | 60.8±10.2 | 62.2±10.7 | 26.6±4.4 | 26.2 ±4.5 | NA | NA |
| Ford et al. RECORD | 5292 | 2649/2643 | 409/2240 | 402/2241 | 77.5±5.6 | 77.4±5.6 | NA | NA | NA | NA |
| Baron et al. | 2259 | 1130/1129 | 0/1130 | 0/1129 | 58.1±7 | 58.0±7 | 28.9±5.0 | 29.1±5.3 | 61.65±19.97 | 60.90±19.97 |
| Jorde et al. | 511 | 256/255 | 161/95 | 153/102 | 62.3±8.1 | 61.9 ±9.2 | 30.1± 4.1 | 29.8± 4.4 | 59.9±21.9 | 61.1±21.2 |
| Scragg et al. | 5108 | 2558/2550 | 1512/1046 | 1457/1093 | 65.9±8.3 | 65.9 ±8.3 | 28.4 ±5.1 | 28.5 ±5.1 | 63.65±23.7 | 62.89±23.46 |
| Shoji et al. J-DAVID | 964 | 488/476 | 301/187 | 277/199 | 65±10.4 | 65±9.67 | Median (IQR) 21.1  (19.0-23.4) | Median (IQR) 21.1 (19.1-23.4) | NA | NA |
| Manson et al. VITAL | 25871 | 12927/12944 | 6380/6547 | 6406/6538 | 67.1±7.0 | 67.1±7.1 | 28.1±5.7 | 28.1±5.8 | NA | NA |
| Aringazina et al. | 336 | 186/150 | 170 /166 | | 56.3 ± 2.4 | | 28.03±1.50 | 27.92±1.33 | 85.59±3.52 | 86.66±3.14 |

NA, not available; STOPIT, Estrogen and Calcitriol in the Prevention of Age-Related Bone Loss; WHI, Women’s Health Initiative clinical trial; IQR, interquartile range; VIDOS, Vitamin D Supplementation in Older Subjects; TIDE, Thiazolidinedione Intervention with vitamin D Evaluation; OPERA, Oral Paricalcitol in Retarding Cardiac Hypertrophy, Reducing Inflammation and Atherosclerosis in Stage 3-5 Chronic Kidney Disease; RECORD, Randomised Evaluation of Calcium Or vitamin D; J-DAVID, Japan Dialysis Active Vitamin D trial; VITAL, The Vitamin D and OmegA-3 Trial
